# Factors associated with knowledge of personal gestational weight gain recommendations

**DOI:** 10.1186/s13104-015-1306-6

**Published:** 2015-08-13

**Authors:** Tracey Ledoux, Patricia Van Den Berg, Patrick Leung, Pamela D Berens

**Affiliations:** University of Houston, 3855 Holman Street, Garrison Gym Rm 104, Houston, TX 77204-6015 USA; Department of Obstetrics and Gynecology, University of Texas Medical Branch, 300 University Blvd, Galveston, TX 77555-0587 USA; University of Houston Graduate College of Social Work, Houston, TX 77204-4013 USA; University of Texas Health Sciences Center, 6431 Fannin St. Suite 3.116, Houston, TX 77030 USA

**Keywords:** Gestational weight gain, Obesity, Institute of Medicine recommendations, Knowledge, Pregnancy

## Abstract

**Objectives:**

Excess adiposity (obesity and excess gestational weight gain, GWG) during pregnancy (EADP) increases risk for gestational diabetes, preeclampsia, and child and maternal obesity. Personal GWG goals predict total GWG. Some estimates suggest only 30% of pregnant women have personal GWG goals that are congruent with Institute of Medicine GWG recommendations. The primary purpose of this study was to determine the extent to which perceived pre-pregnancy weight status, healthcare provider advice, knowledge of EADP risks, and value for healthy GWG predicted knowledge of GWG recommendations. The secondary purpose was to determine sources of GWG information among pregnant women.

**Methods:**

Pregnant women with a confirmed singleton pregnancy completed a one-time survey in obstetric clinic waiting rooms. Logistic regression analysis was used.

**Results:**

246 predominantly African American, low income, overweight/obese women completed surveys. Average age was 25 (SD 5.3) and gestation age ranged from 7 to 40 weeks. Knowledge of pre-pregnancy weight status was the only unique predictor of GWG recommendation knowledge (*B* = .642, *p* = .03). The top three sources of GWG information were physicians, internet, and books. The least frequently reported sources of GWG information were other healthcare providers, community programs, and television.

**Conclusion:**

In low income diverse overweight/obese pregnant women, accurate pre-pregnancy weight status perception was the only significant unique predictor of knowledge of GWG recommendations. Physicians were the preferred source of GWG information. Clinicians should have frequent, ongoing conversations about weight status with women before, during, and after pregnancy.

## Background

The Institute of Medicine (IOM) published gestational weight gain (GWG) guidelines for the first time in 1990 and updated them in 2009 [1]. Per the IOM, optimal GWG ranges depend on pre-pregnancy weight status such that obese women should gain less weight than normal weight women [1]. Exceeding IOM recommendations for GWG increase risks for gestational diabetes, hypertension, preeclampsia, Cesarean delivery, preterm deliveries, neural tube defects, macrosomia, and NICU admissions [2–5]. Not only does pre-pregnancy obesity status compound these risks, but obese women are more likely to exceed GWG recommendations [2–5]. In the United States, more than 60% of women of childbearing age are overweight or obese [6, 7]. Approximately 60% of overweight/obese pregnant women [8] and roughly half of all women exceed IOM recommendations for GWG [9, 10]. The American College of Obstetricians and Gynecologists [11] and the IOM [12] have identified excess adiposity during pregnancy (EADP) as a public health area of concern [13].

Personal GWG goals have been associated with total GWG [14, 15]. Moreover, when personal GWG goals are congruent with IOM GWG recommendations, adherence to IOM recommendations is more likely [16, 17]. Studies have shown 30–79% of pregnant women know how much weight they should gain during pregnancy [18–22]. Identifying factors associated with knowledge of GWG recommendations may explain the variations in these rates and improve the delivery of GWG messages.

Obese women tend to overestimate GWG recommendations [20, 21, 23], probably because there is only modest acknowledgement that GWG recommendations vary depending on pre-pregnancy weight status [24]. There have been mixed results on the relationship between knowledge of pre-pregnancy weight status and GWG [17, 25], but it is unknown whether perceived weight status is related to knowledge of GWG recommendations.

The Health Belief Model posits individuals must value health and acknowledge they are susceptible to a severe health risk before they are motivated to engage in preventive health behaviors [26]. Pregnant women seem to know that EADP is a risk during pregnancy [21, 24, 27], but most cannot identify specific consequences of EADP [21, 24, 27]. Knowing the risks associated with EADP and valuing healthy GWG as a way to mitigate those risks may make women more likely to learn and remember GWG recommendations. According to the Health Belief Model, another important precursor to reducing health risk is a cue to action [26]. External triggers (e.g., advice) may serve as a cue to action. Healthcare provider advice about GWG recommendations should serve as a cue to action among pregnant women.

The primary purpose of this study was to determine whether knowledge of pre-pregnancy weight status, knowledge of EADP risks, perceived value of healthy GWG, and provider advice about GWG recommendations were associated with knowledge of GWG recommendations after controlling for confounding demographic variables. A secondary purpose, was to describe the most common and uncommon sources of GWG information of pregnant women.

## Methods

### Study population and design

In this cross-sectional study, baseline data from a prospective study to examine psychosocial predictors of GWG are reported. Subjects were recruited from prenatal clinic waiting rooms of a university medical center from Fall 2011 through Spring 2013. Eligibility criteria included at least 18 years of age and able and willing to complete a survey in English. All women provided documentation of consent, and all procedures were approved by institutional review boards from the University of Texas Health Science Center and the University of Houston prior to recruitment and enrollment.

### Procedures

A survey packet was constructed using existing measures and items created for this study. An expert panel including psychologists (PVB, TL), dietitians (TL, Kim Matalon, PhD, RDN), a physician (Karen Schneider, MD), and a statistician (PL) reviewed the survey items constructed for this study to ensure content validity. Then items constructed for this study were subjected to cognitive interviews with five pregnant women to ensure comprehension and face validity. Adjustments were made to items based on expert panel and cognitive interview results.

Pregnant women completed survey packets immediately following enrollment, while waiting for a prenatal outpatient appointment. Completion of the questionnaire took 15–20 min. If participants were called into the examination room before they had a chance to complete the questionnaire, they had the opportunity to take it with them and complete it while they waited for their provider in the exam room. Questionnaires along with signed consent forms were returned to office staff or research assistants in the waiting room upon exit.

### Demographic variables

Participants reported age, race, income, education, marital status, gestation age in weeks at the time of the survey, number of prior pregnancies, and pre-pregnancy weight and height. Pre-pregnancy weight and height were used to determine pre-pregnancy body mass index (BMI, kg/m^2^). Pre-pregnancy BMI was used to determine pre-pregnancy weight status (i.e., underweight, normal weight, overweight, or obese) based on IOM weight status cutpoints [1].

### Outcome variable: knowledge of GWG recommendations

Participants indicated, via multiple choice response, the amount of weight they believed they should gain during the current pregnancy to be healthy. The response they selected was compared to IOM recommendations for GWG based on their pre-pregnancy weight status, and participants were categorized as having correct or incorrect knowledge of GWG recommendations.

### Independent variables

*Knowledge of pre*-*pregnancy weight status* was determined by asking participants, “How would you classify your weight just prior to this pregnancy?” Response options were underweight, normal weight, overweight or obese. Responses on this item were compared to actual pre-pregnancy weight status and participants were categorized as having or not having knowledge of pre-pregnancy weight status. This item has been used in previous studies among pregnant women [17, 25].

*EADP risk knowledge* was determined with a 10-item scale [27]. Participants were asked to identify from a list of ten response options (i.e., hypertension, diabetes, Caesarean delivery, large birth weight babies, childhood obesity, fetal growth problems, birth defects, maternal obesity, stillbirth, and premature deliver) which ones were a risk due to EADP. All ten options were risks during pregnancy. Participants received one point for each response option they endorsed. Total scores ranged from 0 to 10 with higher scores indicating greater knowledge about the risks posed to pregnancy by excess adiposity. For this sample, internal consistency was very good with a Cronbach’s alpha of .86.

*Perceived value of healthy GWG* was determined based on a modified version of an item from the Diet and Health Knowledge Survey [28, 29]. Participants were asked, “How important is it to you to have a healthy weight gain (not too much or too little) during your pregnancy?” Response options were very important, somewhat important, not too important, or not at all important. Due to low frequency of reporting somewhat, not too important, and not at all important, these three items were combined so responses were very important or somewhat important.

*Healthcare provider advice about GWG recommendations* was determined with an item constructed for this study. Participants answered with yes or no response to “Has a healthcare provider talked to you about how much weight you should gain during this pregnancy to be healthy?”

### Exploratory variable

Participants were given a list of nine potential sources of GWG information including: family, friend, doctor, other healthcare provider, books, magazines/newspapers, internet, community programs (e.g., WIC, EFNEP, church, school), and television. They were asked to indicate each one they would use for learning about how much weight to gain during pregnancy.

### Statistical analyses

All analyses were conducted using SPSS, version 20 (IBM, Inc., New York, NY). Descriptive analyses were computed for all study variables. Covariates were identified based on those sociodemographic variables (i.e., age, race, marital status, education, employment status, income, pre-pregnancy BMI, and number of previous pregnancies) that were significantly (*p* < .10) related to the dependent variable (i.e., knowledge of GWG recommendations) using univariate analyses for continuous variables and Crosstab Chi Square analyses for group variables. Direct logistic regression was performed to assess the extent to which accuracy of perceived pre-pregnancy weight status, knowledge of EADP, perceived value of healthy GWG, and healthcare provider advice about GWG knowledge was related to knowledge of GWG recommendations. The model was adjusted for significant sociodemographic covariates.

## Results

Three hundred and sixty-three women were given survey packets and 297 (82%) women enrolled. It is unknown why 18% of the survey packets were not returned, but some reasons may be women determining they were not eligible, losing interest in completing the survey, or not having time to complete survey. Of those enrolled, 51 (17%) women were eliminated from the study due to incomplete survey packets. Survey packets with >1 page of missing responses were not included. Compared with the women who were not included in analyses due to missing data, there were no differences on race, ethnicity, income, or education. The final sample consisted of 246 women. Sample characteristics are presented in Table 1. In general, this sample was predominantly African American (58%), overweight or obese (BMI M = 28.5, SD 8.3), and from low socioeconomic conditions (i.e., 49% high school educated or less, 46% earn less than $15,000 per year, and 44% employed). The most commonly reported sources of information about health during pregnancy were doctors (n = 212, 86%), internet (n = 106, 43%), and books (n = 80, 33%). The least reported sources of information about health during pregnancy were community programs like WIC (n = 26, 11%), other healthcare providers (n = 21, 9%), and television (n = 11, 5%). About half the sample (n = 124) reported receiving information from a healthcare provider about GWG during this pregnancy.

**Table 1:** Sample characteristics of participants

Characteristics of participants	Entire sample n = 246 (100%)	Knowledge of GWG recommendations n = 76 (31%)	No knowledge of GWG recommendations n = 170 (69%)
	*Mean* (*SD*) *or n* (*percent*)		
Age (years)	25.8 (5.4)	24.6 (5.2)	26.4 (5.3)
Gestation age (weeks)	23.5 (10.0)	23.9 (8.8)	23.4 (10.5)
Race^a^			
White	47 (19%)	13 (18%)	34 (20%)
Black	143 (58%)	43 (57%)	100 (59%)
Hispanic	63 (26%)	21 (28%)	42 (25%)
Married	163 (66%)	32 (42%)	51 (30%)
Number of prior pregnancies	1.9 (1.7)	1.6 (1.8)	2.1 (1.7)
High school education or less	119 (49%)	34 (45%)	85 (50%)
Employed	107 (44%)	37 (49%)	75 (44%)
Income <$15,000 per year	113 (46%)	37 (49%)	76 (45%)
Pre-pregnancy BMI (kg/m^2^)	28.5 (8.3)	27.7 (8.7)	28.9 (8.1)
Accurate pre-pregnancy weight status perception	116 (47%)	47 (62%)	69 (41%)
EADP risk knowledge (summary score)	3.2 (2.9)	3.3 (2.8)	3.0 (3.0)
Perceived importance of healthy GWG	231 (94%)	72 (94%)	159 (94%)
Healthcare provider advised on GWG	124 (50%)	39 (51%)	85 (50%)

Missing data: income (n = 13, 5%), race (n = 10, 4%), education (n = 3, 1%), employment (n = 5, 2%).

*EADP* excess adiposity during pregnancy, *GWG* gestational weight gain, *BMI* body mass index.

^a^Participants responded with yes/no to all racial groups that applied.

Ninety-four percent (n = 231) said achieving healthy GWG was important to them. Thirty-one percent (n = 76) of the sample had knowledge of GWG recommendations. Sixty-nine percent (n = 170) were incorrect about their pre-pregnancy weight status. The average score on a measure of knowledge of EADP risk was 3/10.

The following covariates were controlled in the final model because they were shown to be significantly (*p* < .10) related to the dependent variable using univariate analyses for continuous variables and Crosstab Chi Square analyses for group variables: age, marital status, and number of previous pregnancies. The model converged, descriptive analyses confirmed meeting logistic regression assumptions, and goodness of fit was confirmed. The full model containing all predictors was statistically significant, *X*^2^ (7, N = 246) = 18.24, *p* = .011, indicating that the model was able to distinguish between respondents who did and did not have knowledge of GWG recommendations. The model as a whole explained between 7.1% (Cox & Snell R square) and 10.1% (Nagelkerke R Square) of the variance in GWG recommendation knowledge, and correctly classified 70.7% of cases. As shown in Table 2, only two independent variables made a unique statistically significant contribution to the model (age and knowledge of pre-pregnancy weight status). The strongest predictor of GWG recommendation knowledge was knowledge of pre-pregnancy weight status (*p* = .03), with an odds ratio of 1.90 (95% CI 1.06, 3.4). Women who knew their pre-pregnancy weight status were 90% more likely to know their personal GWG recommendation. The odds ratio for age was .93 (95% CI .87, 1.0) indicating that for every 1 year increase in age, it would reduce women’s knowledge of GWG recommendations by 7% (*p* = .05).

**Table 2:** Logistic regression predicting knowledge of GWG recommendations

	B	SE	Wald	*p*	OR	95% CI for OR	
						Lower	Upper
Age	−.068	.035	3.855	.050	.934	.873	1.000
Married	.627	.324	3.747	.053	1.872	.992	3.532
Number of prior pregnancies	−.059	.101	.343	.558	.943	.774	1.148
Healthcare provider advice	−.010	.289	.001	.972	.990	.562	1.744
EADP risk knowledge score	−.009	.053	.029	.864	.991	.893	1.100
Perceived value of healthy GWG	−.123	.619	.039	.843	.884	.263	2.977
Accurate perception of pre-pregnancy weight status	.642	.297	4.694	.030	1.901	1.063	3.400

*EADP* excess adiposity during pregnancy, *GWG* gestational weight gain.

## Discussion

In general, knowledge of GWG recommendations was poor among this predominantly African American, overweight/obese sample of pregnant women from low socioeconomic conditions. Only 31% of the participants had knowledge of GWG recommendations. GWG goals have been associated with GWG, so most women in this study are at risk for excess GWG and related complications.

Knowledge of pre-pregnancy weight status was associated with knowledge of GWG recommendations. Women who knew their pre-pregnancy weight status were twice as likely to be knowledgeable about GWG recommendations as women who did not know their pre-pregnancy weight status. One of the Healthy People 2010 goals was for 60% of healthcare providers to routinely discuss preconception counseling with their patients. Perhaps feedback on weight status should be part of preconception counseling.

It is possible there is an underlying quality that makes some women more conscious of their weight status regardless of pregnancy status. Some qualities that may promote greater self-awareness of oneself against an ideal standard are health locus of control, self-efficacy for controlling weight, and conscientiousness among others. Future research should determine mediators and moderators of the association between pre-pregnancy weight status knowledge and GWG recommendation knowledge.

Only two previous studies have examined the association between knowledge of pre-pregnancy weight status and GWG. Herring et al. showed women who misperceived their pre-pregnancy weight were more likely to exceed GWG recommendations than women who knew their pre-pregnancy weight status [17]. Mehta-Lee et al. showed misperceived weight status had no relationship with GWG [25]. Herring’s et al. sample included predominantly white women from middle to upper socioeconomic conditions, while Mehta-Lee’s et al. sample included mostly Hispanic women from low to moderate socioeconomic conditions. This study was the first to show an association between knowledge of pre-pregnancy weight status and knowledge of GWG recommendations among women from low socioeconomic conditions. Future research should determine whether socioeconomic status moderates the associations among knowledge of pre-pregnancy weight status, knowledge of GWG recommendations, and GWG.

Knowledge of EADP risks and perceived importance of healthy GWG were not associated with knowledge of GWG recommendations. Women had little knowledge about specific risks associated with EADP, which is consistent with previous research [18, 21, 24, 27]. Interestingly, 94% of the women said achieving a healthy weight during this pregnancy was very important to them. Other studies have found pregnant women are very interested in nutrition, physical activity, and weight issues [30]. Women may be more concerned with not gaining enough weight than with exceeding GWG recommendations [31]. There has been a significant push by the March of Dimes in response to substantial health disparities in infant mortality rates to encourage women to gain “enough” weight during pregnancy [32]. This would explain the high interest in healthy GWG but low knowledge of EADP risks.

Healthcare provider advice about GWG has influenced patient GWG goals [16, 20, 33] and total GWG [20, 33–36], but more recent studies have not found any association between physician advice and GWG [9, 14]. This may be the first study to find healthcare provider advice about GWG was not related to knowledge of GWG recommendations even though doctors were the most frequently reported source of health information during pregnancy. One issue may be that healthcare providers sometimes give advice that is discrepant from IOM GWG recommendations for a variety of reasons including lack of knowledge or faith in the validity of the recommendations [15, 16, 19, 37, 38]. The content of GWG advice from healthcare providers may interfere with women acquiring knowledge of GWG recommendations.

Most of the women were overweight or obese, putting them at risk. Yet, only half of the participants reported having a discussion with a healthcare provider about GWG, which is consistent with most other studies of patients [9, 14, 16, 18, 19, 27, 30, 39–41] (although some reported higher rates, i.e., 67% [42]). This is in contrast to some provider reports that said 74–95% of providers counseled women on GWG and 76–87% talked with their patients about associated risks [37, 43]. As with many medical issues [44], patients may not always recall conversations about GWG. Alternatively, not all providers consider GWG counseling very important [20, 38], and others do not address it until weight gain becomes an issue during pregnancy [45, 46]. More research is needed on how conversations about GWG between providers and patients unfold and how patients process the information they are given.

Other popular sources of GWG information during pregnancy were the internet and books as was also reported in a recent Canadian study [16]. The least common source of information about health during pregnancy were community programs, other healthcare providers (e.g., nurses, dietitians), and television. In other studies, family and friends [47] and nutritionists from community programs [40] were the top sources of health information during pregnancy. Across all of these studies, the most commonly used sources of GWG information during pregnancy provide easy and instantaneous access to health information. Healthcare providers should recommend evidence based resources in the form of trusted websites and books and include family members and friends in discussions about health behaviors during pregnancy.

Limitations of the current study are the cross-sectional design, which prevents causal associations from being determined, and also that the survey was only offered in English. Self-report data are subject to bias and reporting errors; however, there are no other methods for measuring many of the variables from this study (e.g., knowledge). Pre-pregnancy weight and height were potentially available from sources like patient medical records; however, clinic data are not necessarily more valid sources of weight and height data than patient self-report [48]. Another limitation of this study was the fact that some of the variables were measured with one item, and the wording of the Perceived Value for Healthy GWG item may have led to potential social desirability bias. There are few validated measures of psychosocial constructs for pregnant women. In every case possible, items from existing validated measures (e.g., PRAMS) were used or modified. In addition, cognitive interviews with pregnant women and an expert panel review were conducted with all survey items prior to data collection to ensure content and face validity. Finally, gestation age was not exclusion criteria to participate in this study. Although gestation age was not related to the dependent variable (knowledge of GWG recommendations) responses on the survey regarding independent variables may vary depending on gestation age. The main strength of this study was the characteristics of the sample recruited. This was a low income low education group of predominantly African American women. Excess GWG is highly prevalent in this population. This population is considered underserved with a number of health disparities [49, 50], and this research may serve to improve the quality of health advice and healthcare these women receive during pregnancy.

## Conclusions

Knowledge of GWG recommendations, pre-pregnancy weight status, and adiposity related risks during pregnancy were poor among this sample of predominantly African American pregnant women from low socioeconomic conditions. Knowledge of pre-pregnancy weight status was the only predictor of knowledge of GWG recommendations. Unplanned pregnancies and late and inconsistent access to prenatal care are common problems among pregnant women from impoverished conditions; therefore healthcare providers should have ongoing conversations about weight status and associated risks among all women of childbearing age at each office visit before, during, and after pregnancy. In this study, healthcare provider advice was not associated with knowledge of GWG recommendations, and only about half the sample reported having a conversation with their healthcare provider about GWG. Other studies suggest a number of potential reasons for these findings including providers giving inaccurate and inconsistent GWG advice. Pregnant women from this study and others want GWG advice, and many pregnant women turn to books and the internet for this advice. Future research should identify and test effective methods for delivering GWG advice. Professional and healthcare policy may also promote higher quality clinical practice in this area. Healthcare providers should work with patients to identify the most credible resources to rely on for this information.

## Abbreviations

GWG: gestational weight gain
EADP: excess adiposity during pregnancy
BMI: body mass index
